# Building a mathematical model of the brain

**DOI:** 10.7554/eLife.96231

**Published:** 2024-02-28

**Authors:** Frances Skinner

**Affiliations:** 1 Division of Clinical and Computational Neuroscience, Krembil Brain Institute, University Health Network and Department of Physiology, University of Toronto Toronto Canada

**Keywords:** hippocampus, neuroscience, simulation, neuron, Rodent

## Abstract

Automatic leveraging of information in a hippocampal neuron database to generate mathematical models should help foster interactions between experimental and computational neuroscientists.

**Related research article** Wheeler DW, Kopsick JD, Sutton N, Tecuatl C, Komendantov AO, Nadella K, Ascoli GA. 2024. Hippocampome.org 2.0 is a knowledge base enabling data-driven spiking neural network simulations of rodent hippocampal circuits. *eLife* **12**:RP90597. doi: 10.7554/eLife.90597.

The amount of data that can be gathered about the human brain has been growing exponentially in recent years, but it could be argued that relatively little progress has been made in actually understanding how the brain works. While there may be sociological and philosophical reasons for this lack of progress (see, for example, Thompson, 2021), a main reason is the low level of interactions between the experimental and theoretical/modeling communities in neuroscience (Marder, 2015). Bridging this divide will be difficult because it requires researchers on both sides to leave their comfort zones and learn more about each other’s work, including the constraints that both sides work under. If not, there is a risk that the results of beautiful experiments, or the outputs of thoughtful models, will not be fully appreciated by everyone working in that particular field of neuroscience.

Where does one begin when trying to build a mathematical model of a biological system? In the case of the brain, besides deciding which region of the brain one wants to model and being clear about the goals of the study (Shou et al., 2015), choices need to be made about the level of abstraction. Understanding how the brain works, in both health and disease, requires studying neural circuits at the level of the cell, particularly as neurological diseases are cell-specific (see, for example, Gallo et al., 2020). Furthermore, many studies have made it abundantly clear that circuit function cannot be understood without a greater understanding of the individual cell types making up the circuit (see, for example, Daur et al., 2016 regarding the stomatogastric nervous system). Indeed, when considering a theoretical basis for biology, it is often argued that the correct level of abstraction is the cell (Brenner, 2010).

Hippocampome.org is a database that contains a vast amount of information about the different types of neuronal cells found in the hippocampus – a region of the brain that has major roles in learning and memory – in rodents. The first version of the database contained information on 122 types of neuronal cells based on the shapes of their axons and dendrites, their main neurotransmitters, and various molecular and biophysical properties (Wheeler et al., 2015). Subsequent versions of the database included information on a range of topics including the physiology of the synapses that connect neurons and the electrical behaviour of various neurons.

Now, in eLife, Giorgio Ascoli and colleagues at George Mason University – including Diek Wheeler as first author – present Hippocampome.org v2.0, which enables users to automatically build models that can be used to simulate the electrical behaviour of networks of neurons (Wheeler et al., 2024). Moreover, Hippocampome.org v2.0 includes data and information on over 50 new neuron types. Now, with the click of a button, a user can choose the region (or regions) of the hippocampus they are interested in and the cell types they would like to include in their model, and Hippocampome.org v2.0 will build a model in which the properties of the individual cells and their connections are based on experimental data from multiple research papers. Furthermore, the data come with important metadata (such as the age of the animals), so users can evaluate the values of the various parameters that are included in any model. Indeed, the richness of the data is such that some researchers have been able to make discoveries by applying data-mining techniques to Hippocampome.org (Sanchez-Aguilera et al., 2021).

Deciding how much detail to include in a model is a non-trivial consideration, but it is naturally dependent on the question being asked and the availability of experimental data. Choosing to represent each neuron by a single compartment, rather than including its structure and properties, and using a relatively simple mathematical model called an Izhikevich model (Izhikevich, 2003) to describe the spiking process is both sensible and necessary. Izhikevich models can encompass many, if not all, of the firing properties of biological cells, and although more complex neuron models exist – such as conductance-based models that include ion-channel types – they would make an already complex ‘automated network model building’ challenge even more complex.

With Hippocampome.org v2.0 in hand, it is now possible to start bridging the gap between theory and experiment without having to make a heroic effort to parse the experimental literature. That is, theoretical ’bones’ can be given experimental ‘meat’, as Wheeler et al. demonstrate in simulations of grid cells. Essentially, this resource can be used to bind hypothesis-driven and data-driven modeling (Eriksson et al., 2022).

To truly understand how the brain works, and to help the many individuals suffering from brain disorders, there needs to be stronger collaborations between experimentalists and modellers. This new resource developed by Wheeler et al. provides a practical path towards this outcome.
